# Evaluation of ^134^Ce/^134^La as a PET Imaging Theranostic Pair for ^225^Ac α-Radiotherapeutics

**DOI:** 10.2967/jnumed.122.265355

**Published:** 2023-07

**Authors:** Kondapa Naidu Bobba, Anil P. Bidkar, Niranjan Meher, Cyril Fong, Anju Wadhwa, Suchi Dhrona, Alex Sorlin, Scott Bidlingmaier, Becka Shuere, Jiang He, David M. Wilson, Bin Liu, Youngho Seo, Henry F. VanBrocklin, Robert R. Flavell

**Affiliations:** 1 Department of Radiology and Biomedical Imaging, University of California, San Francisco, San Francisco, California;; 2 Department of Anesthesia, University of California, San Francisco, San Francisco, California;; 3 Department of Radiology and Medical Imaging, University of Virginia, Charlottesville, Virginia;; 4 UCSF Helen Diller Family Comprehensive Cancer Center, San Francisco, California; and; 5 Department of Pharmaceutical Chemistry, University of California, San Francisco, San Francisco, California

**Keywords:** ^134^Ce, ^225^Ac, targeted α-radiotherapy, PET imaging, PSMA-617, YS5 antibody

## Abstract

^225^Ac-targeted α-radiotherapy is a promising approach to treating malignancies, including prostate cancer. However, α-emitting isotopes are difficult to image because of low administered activities and a low fraction of suitable γ-emissions. The in vivo generator ^134^Ce/^134^La has been proposed as a potential PET imaging surrogate for the therapeutic nuclides ^225^Ac and ^227^Th. In this report, we detail efficient radiolabeling methods using the ^225^Ac-chelators DOTA and MACROPA. These methods were applied to radiolabeling of prostate cancer imaging agents, including PSMA-617 and MACROPA-PEG_4_-YS5, for evaluation of their in vivo pharmacokinetic characteristics and comparison to the corresponding ^225^Ac analogs. **Methods:** Radiolabeling was performed by mixing DOTA/MACROPA chelates with ^134^Ce/^134^La in NH_4_OAc, pH 8.0, at room temperature, and radiochemical yields were monitored by radio–thin-layer chromatography. In vivo biodistributions of ^134^Ce-DOTA/MACROPA.NH_2_ complexes were assayed through dynamic small-animal PET/CT imaging and ex vivo biodistribution studies over 1 h in healthy C57BL/6 mice, compared with free ^134^CeCl_3_. In vivo, preclinical imaging of ^134^Ce-PSMA-617 and ^134^Ce-MACROPA-PEG_4_-YS5 was performed on 22Rv1 tumor–bearing male nu/nu-mice. Ex vivo biodistribution was performed for ^134^Ce/^225^Ac-MACROPA-PEG_4_-YS5 conjugates. **Results:** ^134^Ce-MACROPA.NH_2_ demonstrated near-quantitative labeling with 1:1 ligand-to-metal ratios at room temperature, whereas a 10:1 ligand-to-metal ratio and elevated temperatures were required for DOTA. Rapid urinary excretion and low liver and bone uptake were seen for ^134^Ce/^225^Ac-DOTA/MACROPA. NH_2_ conjugates in comparison to free ^134^CeCl_3_ confirmed high in vivo stability. An interesting observation during the radiolabeling of tumor-targeting vectors PSMA-617 and MACROPA-PEG_4_-YS5—that the daughter ^134^La was expelled from the chelate after the decay of parent ^134^Ce—was confirmed through radio–thin-layer chromatography and reverse-phase high-performance liquid chromatography. Both conjugates, ^134^Ce-PSMA-617 and ^134^Ce-MACROPA-PEG_4_-YS5, displayed tumor uptake in 22Rv1 tumor–bearing mice. The ex vivo biodistribution of ^134^Ce-MACROPA.NH_2_, ^134^Ce-DOTA and ^134^Ce-MACROPA-PEG_4_-YS5 corroborated well with the respective ^225^Ac-conjugates. **Conclusion:** These results demonstrate the PET imaging potential for ^134^Ce/^134^La-labeled small-molecule and antibody agents. The similar ^225^Ac and ^134^Ce/^134^La-chemical and pharmacokinetic characteristics suggest that the ^134^Ce/^134^La pair may act as a PET imaging surrogate for ^225^Ac-based radioligand therapies.

## Visual Abstract

**Figure:**
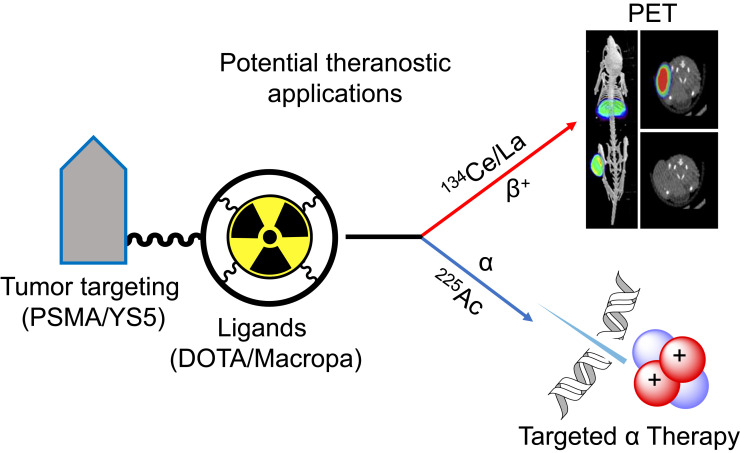

Advances in targeted molecular imaging and radionuclide therapy have given rise to the field of targeted theranostics (1). In this paradigm, a molecular agent with a PET or SPECT imaging isotope (e.g., ^64^Cu, ^89^Zr, or ^123^I) is paired with a cognate radionuclide therapy agent (e.g., ^177^Lu, ^225^Ac, or ^131^I) (2). α-emitting radiotherapies with isotopes, including ^227^Th, ^225^Ac, ^213^Bi, ^212^Pb/^212^Bi, ^211^At, and ^149^Tb, have demonstrated promise in human trials (3*,*^4^). α-particles have a shorter range in tissue (40–100 μm) and higher linear energy transfer than β-particles (5).

To date, ^225^Ac is one of the most promising radionuclides for targeted α-therapy (6). However, an imaging isotope to match with ^225^Ac to measure pharmacokinetics and dosimetry has been elusive (7). Actinium has 2 short-lived daughter isotopes, ^221^Fr and ^213^Bi, that emit low-energy γ-rays, which are challenging to image with SPECT (8). Thus, ^225^Ac therapy is commonly paired with ^68^Ga, ^89^Zr, or ^111^In for imaging-based pharmacokinetic or dosimetry information. However, because of substantial differences in half-life (t_1/2_) (^68^Ga) or chelation chemistry (^89^Zr), these are imperfect PET imaging surrogates for ^225^Ac. To overcome these limitations, lanthanum-based PET imaging agents such as ^132^La (t_1/2_ = 4.8 h, 42% β^+^) and ^133^La (t_1/2_ = 3.9 h, 7% β^+^) have emerged as potential imaging surrogates for ^225^Ac (9*,*^10^). Unfortunately, the t_1/2_ values of these isotopes are considerably shorter than for ^225^Ac, restricting their translation to longer-t_1/2_ macromolecule-based PET imaging.

In this context, the Department of Energy isotope program (11) has recently initiated the production of ^134^Ce, an isotope with a 3.2-d t_1/2_ that decays by electron capture to ^134^La with the emission of low-energy Auger electrons. The ^134^La is a positron emitter (63% β^+^; endpoint energy, 2.69 MeV) with a t_1/2_ of 6.45 min. The unique relationship between the t_1/2_ values of ^134^Ce and ^134^La establishes a secular equilibrium (12). In pioneering work, ^134^Ce cation in the +3 oxidation state has been shown to complex with diethylenetriamine pentaacetate (DTPA) (11) and DOTA (13) and to be used for in vivo PET imaging of the chelate as well as the antibody trastuzumab. It was suggested that the similar chemical characteristics between ^225^Ac^3+^ and ^134^Ce^3+^ and the longer ^134^Ce t_1/2_ (3.2-d) might be advantageous for tracking in vivo pharmacokinetics, especially at later time points. However, DOTA and DTPA require higher molar ratios and elevated temperatures for isotope complexation. Alternatively, MACROPA has demonstrated superior chelate properties for ^225^Ac and a high stability (K_LnL_ = 15.1) for nonradioactive cerium (14), suggesting that it may function well for ^134^Ce/^225^Ac theranostic development (15).

^225^Ac-based radiopharmaceutical therapy has recently attracted great interest in prostate cancer, particularly ^225^Ac-PSMA-617 in small trials, demonstrating great efficacy, especially in the context of resistance to ^177^Lu-PSMA-617 (16*,*^17^). Our own laboratories have identified the antibody YS5, which targets a tumor-selective epitope, CD46, that is highly expressed in prostate cancer (18). An immuno-PET agent, ^89^Zr-DFO-YS5, has successfully imaged both PSMA-positive and PSMA-negative tumor xenografts and patient-derived PDX models (19). Development of cognate ^225^Ac-YS5 radiopharmaceuticals for therapy is currently under way (20–22). These therapeutic approaches would significantly benefit from a companion imaging agent.

Here, we aim to evaluate the potential of positron-emitting ^134^Ce/^134^La as a PET imaging surrogate for ^225^Ac. We describe methods for efficient chelation of ^134^Ce using the MACROPA and DOTA chelators and demonstrate the stability of the conjugates. The imaging and distribution characteristics of the ^134^Ce-labeled tumor-targeting agents PSMA-617 and MACROPA-PEG_4_-YS5 are evaluated in prostate cancer models. These studies demonstrate the feasibility and applicability of ^134^Ce-based radiopharmaceuticals for cancer imaging.

## MATERIALS AND METHODS

### Radiolabeling of DOTA, MACROPA.NH_2_, and PSMA-617 with ^134^CeCl_3_

^134^Ce(NO_3_)_3_ in 0.1 M HCl was produced at the Isotope Production Facility of Los Alamos National Laboratory as previously described (11). Test batches were supplied by the Department of Energy isotope program for our studies. Radiolabeling reactions of DOTA, MACROPA.NH_2_, and PSMA-617 at various ligand-to-metal molar ratios were performed using 2 M NH_4_OAc buffer, pH 8.0, except when the product was used for animal injections (0.1 M NH_4_OAc, pH 8.0). For radiolabeling, aliquots of ^134^CeCl_3_ in 0.1 M HCl (5.17 μL) were mixed with MACROPA.NH_2_ (23 μL, 630 μg/mL in 2 M NH_4_OAc buffer) or DOTA (20 μL, 375 μg/mL in 2 M NH_4_OAc buffer) in 2 M NH_4_OAc buffer, pH 8.0 (100 μL) at 25°C for 30 min and PSMA-617 (1.5 μL, 0.8 μg, 500 μg/mL) at 60°C for 1 h. The reaction solution was analyzed by radio–thin-layer chromatography (TLC) using C_18_ TLC plates (Supelco; Sigma) eluted with 10% NH_4_Cl:MeOH (1:1).

### Radiolabeling of MACROPA-PEG_4_-YS5 with ^134^CeCl_3_

MACROPA-PEG_4_-YS5 (221.4 μg; 1:1 total metal-to-YS5 molar ratio) was incubated with an aliquot of ^134^CeCl_3_ (105 μL, 48.1 MBq) in 2 M NH_4_OAc (pH 8.0) at 25°C for 1 h. The radiolabeling progress was monitored by instant thin-layer chromatography (iTLC) on Varian iTLC silica gel strips using 50 mM ethylenediaminetetraacetic acid, pH 5.5, as an eluent. The reaction mixture was purified over PD10 column gel filtration eluting with 0.9% saline solution.

### Small-Animal PET Imaging

^134^Ce-MACROPA.NH_2_ and ^134^Ce-DOTA reactions in 0.1 M NH_4_OAc buffer were diluted in saline (1:1 ratio), and 4.81–5.92 MBq in 100 μL were administered via the tail vein to 5- to 6-wk-old wild-type C57BL/6 male mice under isoflurane anesthesia. The specific and molar activities were 19.24 GBq/mg and 20.4 GBq/μmol, respectively, for ^134^Ce-MACROPA.NH_2_ and 3.7 GBq/mg and 1.9 GBq/μmol, respectively, for ^134^Ce-DOTA. Dynamic small-animal PET/CT (Inveon; Siemens Medical Solutions) was performed for 1 h simultaneously on 3 mice for both ^134^Ce-MACROPA.NH_2_ and ^134^Ce-DOTA. Free ^134^CeCl_3_ (∼4.81–5.92 MBq) in saline (100 μL) was injected similarly to the method described above, to a group of 2 mice for dynamic small-animal PET/CT and a group of 3 mice for static small-animal PET/CT (20-min PET acquisition) at 2 h and 24 h.

For tumor imaging studies, ^134^Ce-PSMA-617 (∼4.3 MBq) in saline (100 μL) was injected via the tail vein into 22Rv1 tumor–bearing mice, and the mice were imaged at 1 h after injection using small-animal PET/CT. For ^134^Ce-MACROPA-PEG_4_-YS5 (∼4.44 MBq), the conjugate was injected intravenously into mice implanted with 22Rv1 xenografts and imaged at 4 h and then at 1, 2, 4, and 7 d after injection. Small-animal PET/CT was performed with 20 min of PET at earlier time points (4 h, 1d, and 2 d) and with 60 min of PET at later time points (4 and 7 d). The specific and molar activities were 2.58 GBq/mg and 2.67 GBq/μmol, respectively, for ^134^Ce-PSMA-617 and 0.18 GBq/mg and 26.94 GBq/μmol, respectively, for ^134^Ce-MACROPA-PEG_4_-YS5.

## RESULTS

### Radiolabeling of Bifunctional Chelators DOTA and MACROPA.NH_2_

**FIGURE 1.**
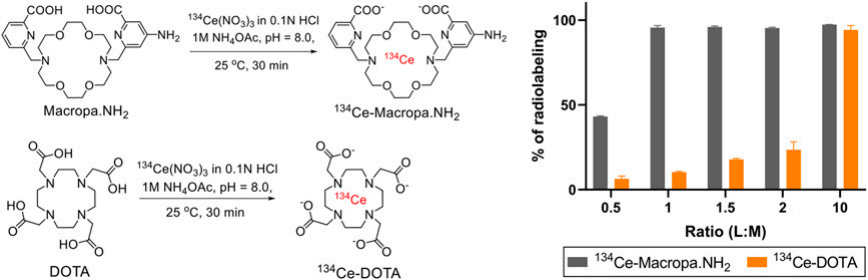
(Left) Radiolabeling of MACROPA.NH_2_ and DOTA with ^134^CeCl_3_. (Right) Percentage radiolabeling at increasing L/M ratios for MACROPA.NH_2_ and DOTA (*n* = 2) at 25 °C, as assayed by radio-TLC.

We assessed the radiolabeling efficiencies of MACROPA.NH_2_ and compared with DOTA at varying ligand-to-metal (L/M) ratios (Fig. 1 left). The L/M ratios were calculated using the stable cerium plus lanthanum present in the ^134^CeCl_3_ solution as per the certificate of analysis (Supplemental Fig. 1; supplemental materials are available at http://jnm.snmjournals.org). As posited, MACROPA.NH_2_ complexed all the ^134^Ce in greater than 95% yield from 0.5:1 to 10:1 L/M ratios. In contrast, DOTA complexed 94.2% ± 1.8% of the ^134^Ce only at the 10:1 L/M ratio (Fig. 1; Supplemental Fig. 2). A slight increase in radiolabeling complexation was observed for DOTA using L/M ratios of 2:1 (32.6% vs. 23.3%) and 5:1 (88.2% vs. 72.53%) at an elevated temperature of 60°C (Supplemental Fig. 3). These studies demonstrate that MACROPA.NH_2_ exhibited a radiolabeling yield superior to that of DOTA, notably allowing rapid, near-quantitative radiolabeling at a 1:1 L/M ratio at room temperature. The ^134^Ce-MACROPA.NH_2_ (1:1 ratio) radiocomplex was analyzed by reverse-phase radio–high-performance liquid chromatography, and the retention time was compared with the ^Nat^Ce-MACROPA.NH_2_ complex (Supplemental Figs. 4–8; Supplemental Scheme 1). However, the radio–high-performance liquid chromatogram showed a tailing behavior, likely due to the ejection of ^134^La from the chelate after the decay by its parent, ^134^Ce. The stability of the ^134^Ce-MACROPA.NH_2_ complex was evaluated in physiologic buffers and in human and rat serum. Over 7 d, more than 95% of the complex was intact in all buffers and serum (Supplemental Fig. 9).

### In Vivo Stability of ^134^Ce-MACROPA.NH_2_ and DOTA Demonstrated by PET Imaging and Biodistribution Studies

**FIGURE 2.**
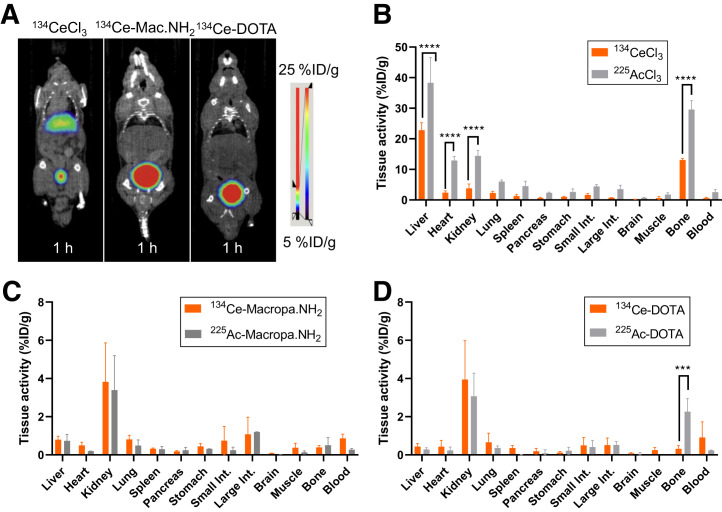
Evaluation of PET imaging of ^134^Ce and chelated complexes in wild-type mouse studies. (A) Coronal small-animal PET/CT images of free ^134^CeCl_3_, ^134^Ce-MACROPA.NH_2_, and ^134^Ce-DOTA in wild-type mice. (B–D) Ex vivo biodistribution of ^134^CeCl_3_ (*n* = 2) and ^225^AcCl_3_ (*n* = 3) (B), ^134^Ce/^225^Ac-MACROPA.NH_2_ (*n* = 3) (C), and ^134^Ce/^225^Ac-DOTA (*n* = 3) (D). Error bars represent SD. ***P < 0.0008. ****P < 0.0001.

After successful ^134^Ce radiolabeling of MACROPA.NH_2_ and DOTA, complex pharmacokinetics and stability were studied in healthy wild-type C57BL/6 mice via PET imaging and biodistribution compared with free ^134^CeCl_3_. ^134^CeCl_3_ showed a gradual increase in liver uptake, as well as in bladder and kidney uptake (Fig. 2A; Supplemental Fig. 10). In contrast, PET imaging of the ^134^Ce-MACROPA.NH_2_ and ^134^Ce-DOTA complexes demonstrated clearance from most organs, with accumulation in the kidneys and bladder at over 1 h after injection, consistent with renal excretion (Fig. 2A; Supplemental Figs. 11–13). The time–activity curves in Supplemental Figure 14 show the slow blood clearance of ^134^CeCl_3_ in comparison with ^134^Ce-MACROPA.NH_2_ and ^134^Ce-DOTA. The 1-h ex vivo biodistribution of ^134^CeCl_3_, ^134^Ce-MACROPA.NH_2_, and ^134^Ce-DOTA are shown in Figures 2B–2D and Supplemental Table 1. High liver (71.5 ± 4.3 percentage injected dose [%ID]/g) and bone (15.54 ± 2.69 %ID/g) uptake was observed for free ^134^CeCl_3,_ with similar results found at 2.5 and 24 h after injection (Supplemental Fig. 15). In contrast, ^134^Ce-MACROPA.NH_2_ (4.36 ± 2.54 %ID/g) and ^134^Ce-DOTA (5.17 ± 2.33 %ID/g) were equally taken up in the kidney, with low accumulation in the liver and other organs, indicating low nonspecific accumulation and renal clearance. Overall, the PET imaging and biodistribution studies of ^134^Ce-MACROPA.NH_2_ and ^134^Ce-DOTA versus free ^134^Ce demonstrated high complex in vivo stability.

The ex vivo biodistribution of ^225^AcCl_3_, ^225^Ac-MACROPA.NH_2_, and ^225^Ac-DOTA (Supplemental Fig. 16; Supplemental Table 2) was assessed and compared with the respective ^134^Ce complexes. Free ^225^Ac accumulates primarily in the liver (38.33 ± 6.75 %ID/g) and bone (29.56 ± 2.40 %ID/g), similarly to ^134^Ce (Fig. 2B). ^225^Ac-MACROPA.NH_2_ (3.54% ± 1.07%) and DOTA (3.07 ± 0.99 %ID/g) complexes displayed a higher uptake in the kidney, with minimal uptake in the liver (0.74 ± 0.19 and 0.28 ± 0.008 %ID/g), similarly to ^134^Ce-MACROPA.NH_2_ and ^134^Ce-DOTA (Figs. 2C and 2D). Notable differences were observed in bone uptake for ^225^Ac-DOTA (2.26 ± 0.56 %ID/g) versus ^134^Ce-DOTA (0.45 ± 0.24 %ID/g) and in blood uptake for ^134^Ce-MACROPA.NH_2_ (0.86 ± 0.19 %ID/g) and ^134^Ce-DOTA (1.07 ± 0.80 %ID/g) versus ^225^Ac-MACROPA.NH_2_ (0.32 ± 0.08 %ID/g) and ^225^Ac-DOTA (0.23 ± 0.02 %ID/g). Taken together, the data indicate that the biodistributions of the ^134^Ce- and ^225^Ac-chelated complexes are largely similar.

### Radiolabeling of Prostate Cancer–Targeting Agents PSMA-617 and MACROPA-PEG_4_-YS5

**FIGURE 3.**
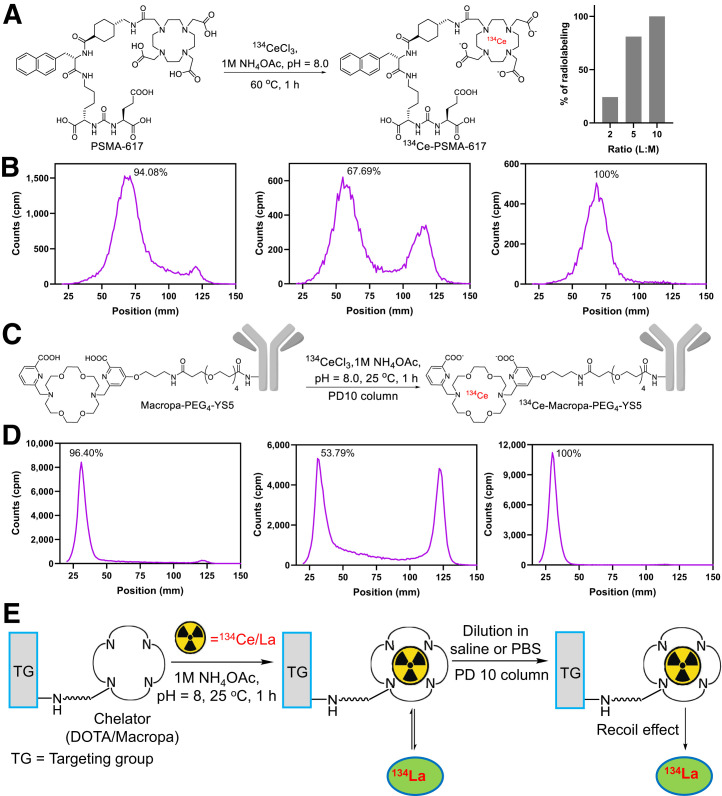
Radiolabeling of prostate cancer–targeting agents PSMA-617 and MACROPA-PEG_4_-YS5. (A) Radiolabeling of PSMA-617 (left) and radiolabeling yields at increasing molar ratios of PSMA-617 (right). (B) Radio-iTLC of ^134^Ce-PSMA-617 (left), same reaction mixture diluted in saline scanned without waiting for 1-h decay (middle), and same radio-iTLC scanned after 1-h decay showing quantitative radiochemical yield (right). (C) Radiolabeling of MACROPA-PEG_4_-YS5. (D) Radio-iTLC of ^134^Ce-MACROPA-PEG_4_-YS5 (left), same reaction mixture after PD10 column purification immediately scanned without waiting for 1-h decay (middle), and same radio-iTLC after 1-h decay (right). (E) ^134^La dechelation due to recoil effect. PBS = phosphate-buffered saline.

Given the encouraging in vivo results in normal mice, we investigated the ^134^Ce radiochemistry of cancer-targeting radiopharmaceuticals, including the small-molecule prostate-specific membrane antigen (PSMA)–targeting agent PSMA-617 (23) and the CD46-targeting antibody derivative MACROPA-PEG_4_-YS5. For PSMA-617, higher L/M ratios were required for quantitative ^134^Ce-labeling, as 24.3%, 81.0%, and 100% radiolabeling yields were noted by radio-TLC for 2:1, 5:1, and 10:1 L/M ratios, respectively (Fig. 3A; Supplemental Fig. 17). The radiolabeling yields were comparable to the similar ratios (10:1) of ^225^Ac-PSMA-617 based on the prior literature (24). After 1 h of incubation of ^134^Ce with PSMA-617 (Fig. 3), iTLC showed 94.1% radiolabeling yield. Surprisingly, the radiolabeling yields were apparently reduced to about 53.2% when the reaction was diluted in saline. However, when the same TLC plate was allowed to decay and rescanned, quantitative labeling was again observed. Similarly, when the apparently 94.1% pure ^134^Ce-PSMA-617 was analyzed on reverse-phase radio–high-performance liquid chromatography (Supplemental Fig. 18), a significant tailing behavior was observed between 4 and 9 min. These data are consistent with the release of ^134^La due to the dechelation or recoil effect after the decay of the parent, ^134^Ce.

On the basis of the favorable model labeling studies, we hypothesized that MACROPA would be a superior chelator to enable ^134^Ce immuno-PET imaging. To facilitate the bioconjugation of MACROPA to the YS5 antibody, we prepared a bifunctional chelator containing MACROPA with a short PEG_4_ linker with an activated TFP ester. MACROPA-PEG_4_-TFP (**7 g**) was synthesized over 7 steps in 56.3% overall yield (Supplemental Figs. 19–37; Supplemental Scheme 2) (25). MACROPA-PEG_4_-TFP ester (**7 g**) was conjugated to lysine residues on YS5 (Supplemental Scheme 3), with an average of about 2.6 chelators per antibody as determined by MALDI-TOF MS (Supplemental Fig. 38). Optimized conditions for MACROPA ^134^Ce-labeling were applied, and the radiochemical yield was 96.4% as confirmed by radio-iTLC, with 69.3% isolated yield after purification and a specific activity of 0.18 GBq/mg (Figs. 3C and 3D). In contrast, DOTA-YS5 was unable to complex ^134^Ce even at higher molar ratios (L/M ratio, 2 or 4) and 40°C (Supplemental Fig. 39). Calculation of the ligand-to-metal ratios was based on the number of chelators per antibody YS5. Unexpectedly, the purified eluted fraction of ^134^Ce-MACROPA-PEG_4_-YS5 showed an apparent decrease in radiochemical purity to about 53.8% (Fig. 3D). As seen in the case of labeled PSMA-617, when the same TLC plate was scanned after decaying for 1 h, 100% radiochemical yield was observed (Fig. 3D). Size-exclusion chromatography demonstrated no evidence of aggregation, whereas an elevated baseline was noticed between the product peak at 9.65 to 25 min, indicating the possible dechelation of daughter isotope ^134^La (Supplemental Fig. 40). The release of daughter ^134^La was also evident when these reaction mixtures were diluted in saline either for purification or for mouse injections, irrespective of MACROPA or DOTA ligands (Fig. 3E).

### In Vitro Analysis and In Vivo Distribution of Prostate-Targeting Agent PSMA-617

**FIGURE 4.**
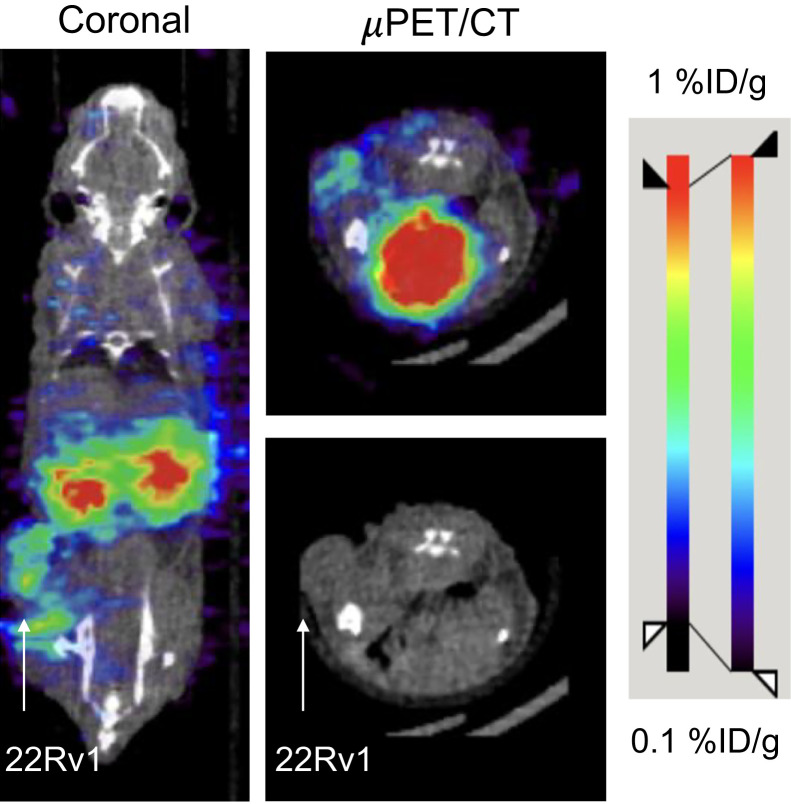
Small-animal PET imaging of ^134^Ce-PSMA-617 in 22Rv1 xenograft at 1 h after injection.

The cell-binding assay of ^134^Ce-PSMA-617 was performed with different concentrations using the 22Rv1 cell line. The percentage of cell-bound activity was significantly higher for all the concentrations than for blocking controls. A decrease in cell-bound activity percentage for a higher concentration (0.8 nM) was observed because of the cold mass effect (Supplemental Fig. 41) (26). Small-animal PET/CT was performed on a 22Rv1 tumor–bearing mouse at 1 h after injection. As shown in Figure 4, most of the activity was in the bladder and kidney at 1 h after injection, with low uptake in the tumor, whereas almost all the activity was eliminated from the other organs. This pattern of tumor uptake is similar to that found using other PSMA-targeting agents in 22Rv1 tumors, which express moderate levels of PSMA (27*,*^28^).

### In Vitro and In Vivo Analysis of ^134^Ce-MACROPA-PEG_4_-YS5

**FIGURE 5.**
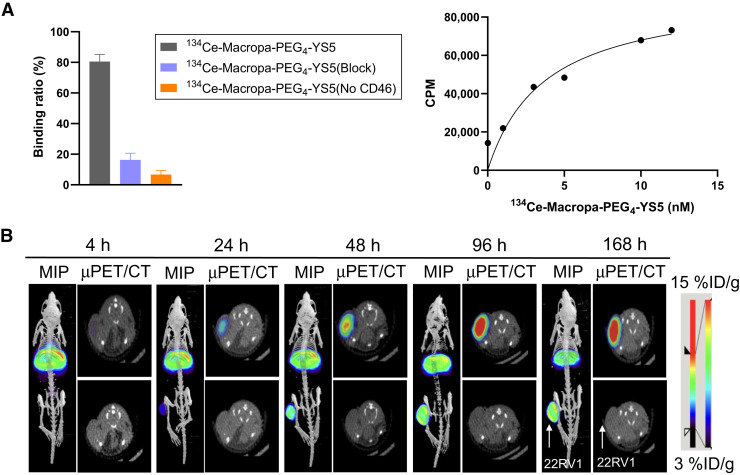
In vitro and in vivo analysis of radioimmunoconjugate ^134^Ce-MACROPA-PEG_4_-YS5. (A) Left: Magnetic bead–based radioligand assay for ^134^Ce-MACROPA-PEG_4_-YS5 (*n* = 3). Right: Saturation binding assay of ^134^Ce-MACROPA-PEG_4_-YS5 on 22Rv1 cells (dissociation constant, 3.7 nM) (*n* = 3). (B) Maximum-intensity-projection PET/CT and transverse small-animal PET/CT images obtained up to 7 d after ^134^Ce-MACROPA-PEG_4_-YS5 injection in mouse bearing 22Rv1 xenografts, demonstrating gradual increase in tumor uptake over time (*n* = 4). MIP = maximum-intensity projection.

The properties of ^134^Ce-MACROPA-PEG_4_-YS5 for immuno-PET imaging of prostate cancer were evaluated. A magnetic bead–based radioligand-binding assay revealed a 80.5% ± 4.6% target binding fraction for ^134^Ce-MACROPA-PEG_4_-YS5 (Fig. 5A), whereas approximately 16.25% ± 4.4% for blocking and approximately 6.7% ± 2.6% for no CD46 were observed (*n* = 3). In a saturation binding assay, the dissociation constant of MACROPA-PEG_4_-YS5 was 3.7 nM, similar to that previously reported for ^89^Zr-DFO-YS5 (6.7 nM) (Fig. 5B) (19). These data demonstrate that ^134^Ce-MACROPA-PEG_4_-YS5 could be synthesized effectively with 1:1 ligand-to-metal ratios, with little or no loss of CD46 binding affinity.

**FIGURE 6.**
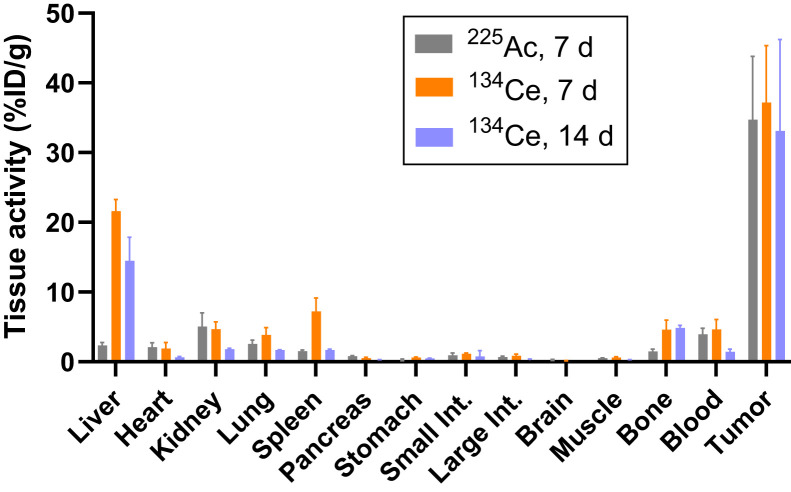
Ex vivo biodistribution analysis of ^134^Ce/^225^Ac-MACROPA-PEG_4_-YS5 in mouse bearing 22Rv1 xenografts at 7 d after injection. Higher tumor and liver uptake was obtained. Error bars represent SD (*n* = 5 at 7 d and 2 at 14 d for ^134^Ce; *n* = 4 for ^225^Ac at 7 d).

Encouraged by the promising radiolabeling studies, we evaluated the PET imaging properties of ^134^Ce-MACROPA-PEG_4_-YS5 in prostate cancer xenografts. Figure 5C and Supplemental Figure 42 show representative small-animal PET/CT images after intravenous administration of ^134^Ce-MACROPA-PEG_4_-YS5 in athymic nude mice bearing 22Rv1 tumors over 7 d. The ex vivo biodistribution confirmed the elevated uptake in the tumor (37.16 ± 8.17 %ID/g) and liver (21.60 ± 1.70 %ID/g). Persistent high tumor uptake (33.11 ± 9.27 %ID/g) was seen 14 d after administration (Fig. 6; Supplemental Table 3).

^225^Ac-MACROPA-PEG_4_-YS5 was radiolabeled, and in vivo biodistribution studies were conducted to compare with the ^134^Ce-labeled YS5 (Supplemental Fig. 43). The imaging and ex vivo biodistribution results for ^134^Ce-MACROPA-PEG_4_-YS5 were similar to those for ^225^Ac-MACROPA-PEG_4_-YS5 for tumor and most tissues (Fig. 6; Supplemental Table 3). High ^225^Ac-MACROPA-PEG_4_-YS5 uptake in the tumor (34.75 ± 9.07 %ID/g) was observed on day 7 after injection, similar to the ^134^Ce-MACROPA-PEG_4_-YS5 uptake (37.16 ± 8.17 %ID/g). However, significant differences in liver (*P* < 0.0001) and spleen (*P* = 0.0109) uptake were observed.

## DISCUSSION

In the design of theranostic agents, it is essential to match the structure and biodistribution of the imaging molecule to that of the radiotherapeutic. Recently, lanthanides have been proposed as nonradioactive surrogates for actinium because of similar chemical properties. ^132^La (t_1/2_ = 4.8 h) and ^133^La (t_1/2_ = 3.9 h) have been studied as complementary PET imaging isotopes for targeted α-therapy with ^225^Ac (t_1/2_ = 9.9 d) (9*,*^10^). Aluicio-Sarduy et al. reported cyclotron-produced ^132^La-labeled alkyl phosphocholine (NM600) in a 4T1 tumor and showed in vivo uptake characteristics similar to those of ^225^Ac (9). Similarly, Nelson et al. described a high-yield cyclotron method to produce ^133^La using natural barium and isotopically enriched ^135^BaCO_3_ targets (10). Potential limitations of ^132^La and ^133^La include shorter t_1/2_ values than for ^225^Ac (t_1/2_ = 9.92 d) and elevated temperatures (80°C–90°C) required for higher radiochemical conversions (>95%). Although these may be more suitable for fast-clearing small molecules, antibody fragments, or small peptides, their t_1/2_ values limit the ability to monitor the pharmacokinetics of macromolecules such as antibodies.

^134^Ce has emerged as an isotope that may be complexed by the same chelates as actinium and thorium. Its decay to ^134^La provides an in situ generator of a positron-emitting isotope with the apparent t_1/2_ of its parent. The pioneering study by Bailey et al. highlighted the cyclotron production of ^134^Ce/^134^La from a natural lanthanum target and established the radiochemistry with ligands DTPA (as a potential surrogate for ^225^Ac) and hydroxypyridinone (as a potential surrogate for ^227^Th) (11). Later, the same group demonstrated the in vivo distribution of ^134^Ce-DOTA-trastuzumab, an internalizing antibody (13). In the present study, imaging and biodistribution of a small-molecule conjugate, PSMA-617, and the antibody YS5 conjugated with MACROPA (MACROPA-PEG_4_-YS5) were conducted on prostate cancer xenografts. Similar tumor uptake was observed between the ^134^Ce- and ^225^Ac-labeled MACROPA-PEG_4_-YS5. The ^134^Ce/^134^La pair allows lengthy in vivo monitoring of molecules because of its extended t_1/2_ of 3.2 d, which is not possible with ^132/133^La radioisotopes.

Broadly speaking, the radiolabeling findings and stability using MACROPA and DOTA chelators with ^134^Ce recapitulate the prior reports using the same chelators with ^225^Ac (15). Radiolabeling efficiency of greater than 95% was achieved with 1:1 ligand-to-metal ratios for MACROPA.NH_2_ and 10:1 for DOTA at room temperature. Dynamic PET imaging and ex vivo biodistribution studies of both ^134^Ce-MACROPA.NH_2_ and ^134^Ce-DOTA confirm in vivo stability and a biodistribution similar to that of ^225^Ac-MACROPA.NH_2_ and DOTA complexes. Overall, the radiolabeling methodologies show that MACROPA.NH_2_ was more efficient than DOTA and that both complexes showed excellent overall stability.

After radiolabeling and purification into saline of the tumor-targeting agents PSMA-617 and MACROPA-PEG_4_-YS5 for mouse administration, we chromatographically observed the release of the daughter radionuclide ^134^La from the chelate. In the reaction mixture, before dilution or purification, the ^134^La may be rechelated after recoil effect if excess ligand is present (Fig. 3E). However, the rechelation may not occur in vivo even if the excess ligand is present, leading to possible ^134^La redistribution. Though the stability constants were high for ^Nat^La-MACROPA (14.91) and ^Nat^Ce-MACROPA (15.11) (14), the ^134^Ce bond dissociation occurs because of the nuclear recoil effect through electron capture decay and subsequent Auger electron emission (29). A similar phenomenon was seen by Severin et al. for another in vivo PET generator, ^140^Nd (t_1/2_ = 3.4 d, Electron capture (EC)/^140^Pr (t_1/2_ = 3.4 m, β^+^), with DOTA-LM3 (small peptide) and DTPA-ATN 291 (antibody). In their work, small differences in tissue distribution were noted via pre- and postmortem imaging—differences that were attributed to redistribution of the daughter. The differences were greater for noninternalizing agents (30*,*^31^). Our imaging findings are also consistent with these prior reports.

The imaging properties of ^134^Ce/^134^La have been evaluated in prostate cancer models using PSMA-617 and MACROPA-PEG_4_-YS5. Low to moderate tumor uptake of ^134^Ce-PSMA-617 was observed at 1 h after administration. High kidney uptake of PSMA-based targeting vectors is known, as they tend to excrete through renal elimination and the mouse kidneys express PSMA (27*,*^28^). In contrast, ^134^Ce-MACROPA-PEG_4_-YS5 showed elevated tumor uptake. Our findings are consistent with our prior report demonstrating elevated uptake of ^89^Zr-DFO-YS5, compared against ^68^Ga-PSMA-11 in the 22Rv1 xenograft model (19).

Remarkably, biodistribution studies of ^134^Ce-MACROPA-PEG_4_-YS5 showed tissue distribution almost identical to that of ^225^Ac-MACROPA-PEG_4_-YS5 except for the liver and spleen. The high liver uptake observed in early images at 24 h (Fig. 5B) may be due to redistribution of daughter ^134^La after ejection from the chelate. This possibility will be further investigated in future studies by conducting pre- and postmortem imaging and comparing it with ^225^Ac more systematically.

One notable advantage to using ^134^Ce is that it allows facile imaging of conjugates bearing the MACROPA chelate, which was previously limited to therapeutic radionuclides. The similar chemical properties of these radionuclides (^134^Ce/^225^Ac) may allow a single molecular platform by complexing with the ligands DOTA or MACROPA. This complexation could facilitate predicting the tumor distribution of ^225^Ac-labeled targeting vectors (^225^Ac-PSMA-617 or MACROPA-PEG_4_-YS5) based on the (^134^Ce-PSMA-617 or MACROPA-PEG_4_-YS5) PET imaging results. Hence, this methodology addresses an important challenge in radiopharmaceutical sciences, namely the study of the biodistribution of ^225^Ac radiopharmaceuticals. Overall, these studies support our premise that ^134^Ce/^134^La may serve as an imaging radionuclide to pair with ^225^Ac.

## CONCLUSION

MACROPA.NH_2_ showed exceptional radiolabeling efficiency with ^134^Ce at room temperature. PET imaging of ^134^Ce-MACROPA.NH_2_ and ^134^Ce-DOTA revealed that both tracers are highly stable in vivo. The ex vivo biodistributions of both ^134^Ce-DOTA and MACROPA.NH_2_ were almost identical to the respective ^225^Ac complexes. ^134^Ce-PSMA-617 shows high binding affinity and uptake in prostate cancer 22Rv1 xenografts. A bifunctional analog for MACROPA was synthesized, conjugated with antibody YS5, and radiolabeled with ^134^Ce and ^225^Ac. Both the PET imaging and the biodistribution of ^134^Ce-MACROPA-PEG_4_-YS5 demonstrate elevated tumor retention in 22Rv1 prostate cancer xenografts. The ex vivo biodistribution is consistent with the ^225^Ac-MACROPA-PEG_4_-YS5 distribution in most tissues, including the tumor. These studies support the future development of ^134^Ce-radiopharmaceuticals for cancer imaging as a companion to paired α-particle radiotherapeutics.

## DISCLOSURE

Kondapa Naidu Bobba and Robert Flavell have filed a patent application, “Radioimmunoconjugates and Therapeutic Uses Thereof” provisional patent application number 63/344537. This study was supported by U.S. Department of Energy, Office of Science, Office of Isotope R&D and Production, DOE Isotope program under Award Number DE-SC-0023467 and Department of Defense grant W81XWH2110792. No other potential conflict of interest relevant to this article was reported.

## Notes

KEY POINTS **QUESTION:** Are the radiochemistry and in vitro/in vivo characteristics of ^134^Ce/^134^La chelates similar to those of ^225^Ac? **PERTINENT FINDINGS:** ^134^Ce/^134^La efficiently forms stable complexes with ^225^Ac-chelates, DOTA, and MACROPA. These may allow a single molecular platform for imaging and radiotherapy. The ex vivo tissue biodistribution was largely similar between ^225^Ac- and ^134^Ce-labeled antibody YS5, with the exception of liver and spleen. **IMPLICATIONS FOR PATIENT CARE:** Identification of an imaging surrogate for ^225^Ac may aid in the development of targeted α-radiotherapeutics and enable visualization of their distribution. Imaging with ^134^Ce-labeled radiopharmaceuticals may guide therapeutic dosing of the concomitant ^225^Ac-labeled molecule.
